# Supplementary material for: Stepwise wiring of the Drosophila olfactory map requires specific Plexin B levels
Source: eLife. 2018 Aug 23;7:e39088. doi: 10.7554/eLife.39088 (PMC6118820; doi:10.7554/eLife.39088)
Supplement: Supplementary file 1. [file elife-39088-supp1.docx]

**Supplementary File 1.** Genotypes of flies in each experiment.

| **Figure** | | **Genotype** |
| --- | --- | --- |
| ***Figure 1*** | | |
| A | *Pebbled-GAL4, UAS-mtdTomato/+ (or Y)* | |
| B | *Pebbled-GAL4, UAS-mtdTomato/+ (or Y);;; plexB^–^/plexB^–^* | |
| C | Control*: Pebbled-GAL4, UAS-mtdTomato/+ (or Y)*  *plexB^–/–^: Pebbled-GAL4, UAS-mtdTomato/+ (or Y);;; plexB^–^/plexB^–^* | |
| E | *UAS-mCD8-GFP/+;; PlexB-GAL4/+* | |
| F | *eyFLP, UAS-FRT-Stop-FRT-mCD8-GFP/+ (or Y);;; PlexB-GAL4/+* | |
| G | *Peb-GAL4: Pebbled-GAL4, UAS-mtdTomato/+ (or Y)*  *PlexB-GAL4∩eyFLP: eyFLP, UAS-FRT-Stop-FRT-mCD8-GFP/+ (or Y);;; PlexB-GAL4/+* | |
| I | *PlexB-Tag* | |
| J | *eyFLP/+ (or Y);;; PlexB-Tag* | |
| K | *PlexB-Tag* | |
| L | *eyFLP/+ (or Y);;; PlexB-Tag* | |
| M | ORN axons*: Pebbled-GAL4, UAS-mtdTomato/+ (or Y)*  PlexB-V5 in ORN axons*: eyFLP/+ (or Y);;; PlexB-Tag* | |
| ***Figure 1-figure supplement 1*** | | |
| A | *PlexB-Tag* | |
| B | *+/+: Pebbled-GAL4, UAS-mtdTomato/+ (or Y)*  *PlexB-Tag/+: Pebbled-GAL4, UAS-tdTomato/+ (or Y);;; PlexB-Tag/+*  *+/plexB^–^: Pebbled-GAL4, UAS-tdTomato/+ (or Y);;; +/plexB^–^*  *PlexB-Tag/plexB^–^: Pebbled-GAL4, UAS-tdTomato/+ (or Y);;; PlexB-Tag/plexB^–^* | |
| C | *+/+: Pebbled-GAL4, UAS-mtdTomato/+ (or Y)*  *Tag/+: Pebbled-GAL4, UAS-tdTomato/+ (or Y);;; PlexB-Tag/+*  *+/–: Pebbled-GAL4, UAS-tdTomato/+ (or Y);;; +/plexB^–^*  *Tag/–: Pebbled-GAL4, UAS-tdTomato/+ (or Y);;; PlexB-Tag/plexB^–^* | |
| D | *UAS-RedStinger(NLS-DsRed)/+; PlexB-GAL4/+* | |
| ***Figure 2*** | | |
| A | *Pebbled-GAL4, UAS-mCD8-GFP/+ (or Y)* | |
| B | *Pebbled-GAL4, UAS-mCD8-GFP/+ (or Y); UAS-PlexB/+* | |
| C | *plexB^–/–^: Pebbled-GAL4, UAS-mtdTomato/+ (or Y);;; plexB^–^/plexB^–^*  Control*: Pebbled-GAL4, UAS-mCD8-GFP/+ (or Y)*  PlexB OE 25°C*:* *Pebbled-GAL4, UAS-mCD8-GFP/+ (or Y); UAS-PlexB/+* (25°C)  PlexB OE 29°C*:* *Pebbled-GAL4, UAS-mCD8-GFP/+ (or Y); UAS-PlexB/+* (29°C) | |
| D | *Or67d-QF, QUAS-mCD8-GFP/+; UAS-PlexB-RNAi/+* | |
| E | *Or67d-QF, QUAS-mCD8-GFP/Pebbled-GAL4; UAS-PlexB-RNAi/+* | |
| F | Control*: Or67d-QF, QUAS-mCD8-GFP/+; UAS-PlexB-RNAi/+*  PlexB-RNAi*: Or67d-QF, QUAS-mCD8-GFP/Pebbled-GAL4; UAS-PlexB-RNAi/+* | |
| ***Figure 3*** | | |
| A | *Pebbled-GAL4, UAS-mCD8-GFP/+ (or Y)* | |
| B | *Pebbled-GAL4, UAS-mCD8-GFP/+ (or Y)* | |
| C | *eyFLP, UAS-FRT-Stop-FRT-mCD8-GFP/+ (or Y);;; PlexB-GAL4/+* | |
| D | *eyFLP/+ (or Y);;; PlexB-Tag* | |
| E | *eyFLP/+ (or Y);;; PlexB-Tag* | |
| ***Figure 3-figure supplement 1*** | | |
| A | *UAS-RedStinger(NLS-DsRed)/+; PlexB-GAL4/+* | |
| B | *eyFLP/+ (or Y);;; PlexB-Tag* | |
| C | *eyFLP/+ (or Y);;; PlexB-Tag* | |
| ***Figure 4*** | | |
| A | *Pebbled-GAL4, UAS-dcr2/+ (or Y); Or92a-rCD2/+* | |
| B | *Pebbled-GAL4/+ (or Y); Or92a-rCD2/UAS-PlexB* | |
| C | *Pebbled-GAL4/+ (or Y); Or92a-rCD2/UAS-PlexB* | |
| D | *Pebbled-GAL4/+ (or Y); Or92a-rCD2/UAS-PlexB* | |
| E | *Pebbled-GAL4, UAS-dcr2/+ (or Y); Or92a-rCD2/UAS-PlexB-RNAi* | |
| F | *Pebbled-GAL4, UAS-dcr2/+ (or Y); Or92a-rCD2/UAS-PlexB-RNAi* | |
| G | *Pebbled-GAL4, UAS-dcr2/+ (or Y); Or92a-rCD2/UAS-PlexB-RNAi* | |
| ***Figure 4-figure supplement 1*** | | |
| A | *AM29-GAL4, UAS-mCD8-GFP/+* | |
| B | *AM29-GAL4, UAS-mCD8-GFP/UAS-PlexB* | |
| C | *AM29-GAL4, UAS-mCD8-GFP/UAS-PlexB* | |
| D | *AM29-GAL4, UAS-mCD8-GFP/UAS-PlexB* | |
| E | *AM29-GAL4, UAS-mCD8-GFP/+;; plexB^–^/plexB^–^* | |
| F | *AM29-GAL4, UAS-mCD8-GFP/+;; plexB^–^/plexB^–^* | |
| G | *AM29-GAL4, UAS-mCD8-GFP/+;; plexB^–^/plexB^–^* | |
| ***Figure 5*** | | |
| A | *Pebbled-GAL4, UAS-mtdTomato/+ (or Y)* | |
| B | *Pebbled-GAL4, UAS-mtdTomato/+ (or Y); sema2b^–^/+* | |
| C | *Pebbled-GAL4, UAS-mtdTomato/+ (or Y); UAS-PlexB/+* | |
| D | *Pebbled-GAL4, UAS-mtdTomato/+ (or Y); sema2b^–^/UAS-PlexB* | |
| E | *+/+: Pebbled-GAL4, UAS-mtdTomato/+ (or Y)*  *Sema2b^–^/+: Pebbled-GAL4, UAS-mtdTomato/+ (or Y); sema2b^–^/+*  PlexB OE_*+/+: Pebbled-GAL4, UAS-mtdTomato/+ (or Y); UAS-PlexB/+*  PlexB OE_ *Sema2b^–^/+: Pebbled-GAL4, UAS-mtdTomato/+ (or Y); sema2b^–^/UAS-PlexB* | |
| F | *Pebbled-GAL4, UAS-mtdTomato/+ (or Y)* | |
| G | *Pebbled-GAL4, UAS-mtdTomato/+ (or Y); UAS-PlexB-RNAi/+* | |
| H | *Pebbled-GAL4, UAS-mtdTomato/+ (or Y); sema2b^–^/sema2b^–^* | |
| I | *Pebbled-GAL4, UAS-mtdTomato/+ (or Y); UAS-PlexB-RNAi, sema2b^–^/sema2b^–^* | |
| J | Control*: Pebbled-GAL4, UAS-mtdTomato/+ (or Y)*  PlexB-RNAi*: Pebbled-GAL4, UAS-mtdTomato/+ (or Y); UAS-PlexB-RNAi/+*  *Sema2b_*Control*: Pebbled-GAL4, UAS-mtdTomato/+ (or Y); sema2b^–^/sema2b^–^*  *Sema2b^–/–^_*PlexB-RNAi*: Pebbled-GAL4, UAS-mtdTomato/+ (or Y); UAS-PlexB-RNAi, sema2b^–^/sema2b^–^* | |
| K | PlexB OE*: Pebbled-GAL4/+ (or Y); Or92a-rCD2/UAS-PlexB*  *2b^–^/+,* PlexB OE*: Pebbled-GAL4/+ (or Y); Or92a-rCD2/UAS-PlexB, sema2b^–^* | |
| L | PlexB OE*: Pebbled-GAL4/+ (or Y); Or92a-rCD2/UAS-PlexB*  *2b^–^/+,* PlexB OE*: Pebbled-GAL4/+ (or Y); Or92a-rCD2/UAS-PlexB, sema2b^–^* | |
| M | PlexB OE*: Pebbled-GAL4/+ (or Y); Or92a-rCD2/UAS-PlexB*  *2b^–^/+,* PlexB OE*: Pebbled-GAL4/+ (or Y); Or92a-rCD2/UAS-PlexB, sema2b^–^* | |
| ***Figure 5-figure supplement 1*** | | |
| A | *Pebbled-GAL4, UAS-mCD8-GFP/+ (or Y)* | |
| B | PlexB OE*: AM29-GAL4, UAS-mCD8-GFP/UAS-PlexB*  *2b^–^/+,* PlexB OE*: AM29-GAL4, UAS-mCD8-GFP/UAS-PlexB, sema2b^–^* | |
| C | PlexB OE*: AM29-GAL4, UAS-mCD8-GFP/UAS-PlexB*  *2b^–^/+,* PlexB OE*: AM29-GAL4, UAS-mCD8-GFP/UAS-PlexB, sema2b^–^* | |
| D | PlexB OE*: AM29-GAL4, UAS-mCD8-GFP/UAS-PlexB*  *2b^–^/+,* PlexB OE*: AM29-GAL4, UAS-mCD8-GFP/UAS-PlexB, sema2b^–^* | |
